# *SPP1* as a Potential Stage-Specific Marker of Colorectal Cancer

**DOI:** 10.3390/cancers17193200

**Published:** 2025-09-30

**Authors:** Eva Turyova, Peter Mikolajcik, Michal Kalman, Dusan Loderer, Miroslav Slezak, Maria Skerenova, Emile Johnston, Tatiana Burjanivova, Juraj Miklusica, Jan Strnadel, Zora Lasabova

**Affiliations:** 1 Department of Molecular Biology and Genomics, Jessenius Faculty of Medicine and Martin University Hospital, 036 01 Martin, Slovakia; eva.turyova@uniba.sk (E.T.); tatiana.burjanivova@uniba.sk (T.B.); 2 Clinic of Surgery and Transplant Center, Jessenius Faculty of Medicine in Martin, Comenius University in Bratislava, Martin University Hospital, 036 01 Martin, Slovakia; peter.mikolajcik@uniba.sk (P.M.);; 3 Department of Pathological Anatomy, Jessenius Faculty of Medicine in Martin and Comenius University in Bratislava, Martin University Hospital, 036 01 Martin, Slovakia; 4 Biomedical Center in Martin, Jessenius Faculty of Medicine, Martin University Hospital, 036 01 Martin, Slovakia; 5 Technische Universität Wien, 1060 Vienna, Austria

**Keywords:** colorectal cancer, metastases, differential gene expression, hypoxia, EMT

## Abstract

**Simple Summary:**

Colorectal cancer is the third most common type of cancer and is often diagnosed at an advanced stage. In addition, the high heterogeneity of this disease leads to complications in the diagnostic process, prognosis evaluation, and the development of new treatment strategies. For this reason, it is essential to search for new diagnostic and prognostic markers that would allow for earlier detection and more accurate assessments of patient outcomes. Our goal is to identify genes that are differentially expressed in colorectal cancer and that may play a role in its initiation, progression, and metastasis, with potential for future use as biomarkers or therapeutic targets for the disease.

**Abstract:**

**Background:** Colorectal cancer is the third most diagnosed cancer and a leading cause of cancer-related deaths worldwide. Early detection significantly improves patient outcomes, yet many cases are identified only at late stages. The high molecular and genetic heterogeneity of colorectal cancer presents major challenges in accurate diagnosis, prognosis, and therapeutic stratification. Recent advances in gene expression profiling offer new opportunities to discover genes that play a role in colorectal cancer carcinogenesis and may contribute to early diagnosis, prognosis prediction, and the identification of novel therapeutic targets. **Methods:** This study involved 142 samples: 84 primary tumor samples, 27 liver metastases, and 31 adjacent non-tumor tissues serving as controls. RNA sequencing was performed on a subset of tissues (12 liver metastases and 3 adjacent non-tumor tissues) using a targeted RNA panel covering 395 cancer-related genes. Data processing and differential gene expression analysis were carried out using the DRAGEN RNA and DRAGEN Differential Expression tools. The expression of six genes involved in hypoxia and epithelial-to-mesenchymal transition (EMT) pathways (*SLC16A3*, *ANXA2*, *P4HA1*, *SPP1*, *KRT19*, and *LGALS3*) identified as significantly differentially expressed was validated across the whole cohort via quantitative real-time PCR. The relative expression levels were determined using the ΔΔct method and log2FC, and compared between different groups based on the sample type; clinical parameters; and mutational status of the genes *KRAS*, *PIK3CA*, *APC*, *SMAD4*, and *TP53*. **Results:** Our results suggest that the expression of all the validated genes is significantly altered in metastases compared to non-tumor control samples (*p* < 0.05). The most pronounced change occurred for the genes *P4HA1* and *SPP1*, whose expression was significantly increased in metastases compared to non-tumor and primary tumor samples, as well as between clinical stages of CRC (*p* < 0.001). Furthermore, all genes, except for *LGALS3*, exhibited significantly altered expression between non-tumor samples and samples in stage I of the disease, suggesting that they play a role in the early stages of carcinogenesis (*p* < 0.05). Additionally, the results suggest the mutational status of the *KRAS* gene did not significantly affect the expression of any of the validated genes, indicating that these genes are not involved in the carcinogenesis of *KRAS*-mutated CRC. **Conclusions:** Based on our results, the genes *P4HA1* and *SPP1* appear to play a role in the progression and metastasis of colorectal cancer and are candidate genes for further investigation as potential biomarkers in CRC.

## 1. Introduction

Colorectal cancer (CRC) is the third most common malignant disease worldwide, and is often diagnosed in its late stages; after lung cancer, it was the second leading cause of cancer death in 2022 (9.3%) [1,2]. New CRC cases are projected to reach as many as 3.2 million globally in 2040 [3]. CRC is a neoplastic disease with a genetic background, exhibiting enormous heterogeneity, which is attributed to the diverse pathways of its development, as well as the involvement of numerous genes and molecular pathways, leading to the formation of carcinomas with distinct histological and molecular features [4,5,6,7]. The varying behavior of cancer cells, driven by their underlying genetic and molecular profiles, ultimately complicates the diagnostic process, prognosis evaluation, treatment optimization, and the development of new therapeutic strategies.

The prognosis of CRC is significantly influenced by the presence of metastases, regardless of their location. The most common site for metastasis in both colon and rectal cancer is the liver [8]. However, the metastatic pattern differs based on the histological subtype and the localization of the primary tumor [8,9,10]. Metastasis is complex and involves the engagement of numerous genes and signaling pathways. Although many details remain unclear, the hypoxia and epithelial-to-mesenchymal transition (EMT) pathways are closely linked to metastasis. Hypoxia is a common characteristic of many solid tumors that triggers numerous downstream signaling pathways, resulting in proliferation, altered cellular metabolism, and angiogenesis, which can lead to higher invasiveness and a tendency to metastasize [11]. Hypoxia induces EMT, during which epithelial cells acquire a mesenchymal phenotype and invasive potential. EMT also allows cells to adapt to the changing tumor microenvironment and metastasize [12]. Both processes are accompanied by numerous changes, whether in the cell cytoskeleton or in the expression of genes and proteins involved in these events [13].

Despite enormous efforts, there is still no completely effective therapy for advanced metastatic CRC, leading to a large number of deaths related to advanced disease. The complex and multifaceted nature of CRC, including its metastasis, underscores the urgent need for reliable biomarkers that can facilitate earlier diagnosis, more precise prognosis, and the development of targeted therapies. Therefore, in our work, we focused on identifying the differential expression of genes related to hypoxia and EMT in metastatic CRC, with the aim of discovering new potential biomarkers for diagnosis, prognosis assessment, and treatment strategies in patients with advanced CRC. The primary aim of this study was to identify hypoxia- and EMT-related genes that are differentially expressed between colorectal cancer liver metastases and adjacent non-tumor colon tissue through the targeted RNA sequencing of the cancer transcriptome. We further assessed whether these expression patterns differed by KRAS mutational status. Through subsequent validation, we analyzed the expression profiles of six key genes *(SLC16A3*, *ANXA2*, *P4HA1*, *SPP1*, *KRT19*, and *LGALS3*) in a cohort of CRC patients, investigating their potential as biomarkers in CRC.

## 2. Materials and Methods

### 2.1. Patients and Clinical Tissue Samples

The entire cohort included 113 patients with colorectal cancer or metastatic colorectal cancer. Samples were obtained from primary tumors of various clinical stages (n = 84), adjacent non-tumor colon tissue (n = 31), and liver metastases (n = 27). Among seven primary tumor samples classified as stage IV, three had extrahepatic metastases (lung, peritoneum, bone). These were retained to capture the molecular features of advanced CRC, regardless of metastatic site. No matched primary/metastasis pairs were available, and the non-tumor tissue samples were collected from patients with primary CRC. All the samples were obtained in collaboration with the University Hospital Martin, the Clinic of General, Visceral and Transplant Surgery, and the Department of Pathological Anatomy (Martin, Slovakia). Tumor tissue surgical excisions were immersed in a solution of Dulbecco’s modified Eagle’s medium, fetal bovine serum (10%), and penicillin/streptomycin and stored at 4 °C. After delivery at the Department of Molecular Biology and Genomics, the tissues were transferred into RNA later (QIAGEN, Hilden, Germany) and stored at −80 °C until RNA isolation.

The inclusion criteria were confirmation of diagnosis by histopathological examination, TNM classification, and clinical stage. If a patient sample corresponded to a primary tumor with clinical stage I–IV, the patient was included in the primary tumor patient group. If a patient sample corresponded to a metastatic tumor with clinical stage IV, the patient was included in the metastatic (liver metastasis) patient group. The exclusion criteria included simultaneous presence of other cancer types, liver metastases with unknown origin, and age under 40 years. Histopathological evaluation, including tumor staging, grading, and typing, was performed by experienced pathologists. The clinicopathological characteristics of the patients included in the study are listed in Table 1. This study was approved by the Ethics Committee of the Jessenius Faculty of Medicine in Martin, Comenius University in Bratislava, under decision number 1863/2016.

Tumor-infiltrating lymphocytes (TILs) and peritumoral lymphoid reactions (PTILs) were histologically evaluated by experienced pathologists based on standard criteria. Briefly, TILs were evaluated in 10 or more high-power fields (HPFs) and were considered positive when five or more intraepithelial lymphocytes were present in at least one HPF at 40× magnification. The PTIL was assessed in two forms: ‘Crohn like’ and ’lichenoid’. The presence of at least one of these two features was considered indicative of a positive PTIL [14,15]. Microsatellite instability (MSI) status was assessed using a pentaplex PCR assay targeting five mononucleotide markers (BAT25, BAT26, NR21, NR24, and NR27), as described by us [16]. The PCR products were analyzed by capillary electrophoresis, and samples were classified as MSI-high if instability was observed in two or more markers, or microsatellite stable (MSS) if no instability was detected. If instability was detected in one marker, the sample was classified as MSI-low. The mutational status of five key colorectal cancer driver genes—*KRAS*, *PIK3CA*, *SMAD4*, *APC*, and *TP53*—was determined using a combination of next-generation sequencing (NGS) and PCR-based diagnostics. NGS was performed as described by us [17], utilizing the AmpliSeq for Illumina Cancer Hotspot Panel v2, which covers common mutational hotspots. DNA was extracted from fresh surgical samples or formalin-fixed paraffin-embedded (FFPE) tumor tissues, libraries were prepared, and sequencing was conducted on the MiSeq platform according to the manufacturer’s instructions. In some cases, the mutational status of the *KRAS* gene was evaluated using the cobas^®^ 4800 *KRAS* Mutation Test (Roche Molecular Systems, Branchburg, NJ, USA), which detects mutations in codons 12, 13, and 61. All molecular results were obtained for clinical purposes and were part of the clinical data that were available for analysis in this study with institutional approval and approved by the ethics committee.

### 2.2. RNA Purification and cDNA Preparation

Total RNA was extracted using the RNeasy Mini Kit (QIAGEN, Hilden, Germany) according to the manufacturer’s instructions, treated with DNase (QIAGEN, Hilden, Germany), and stored a −80 °C. The purified RNA was quantified using the QUBIT 3.0 Fluorometer (Thermo Fisher Scientific, Waltham, MA, USA), and the RNA quality was assessed on an Agilent Bioanalyzer 2100 (Agilent Technologies, Santa Clara, CA, USA). The total RNA was reverse transcribed according to the manufacturer’s instructions using a High-Capacity cDNA Reverse Transcription Kit with RNase inhibitor (Thermo Fisher Scientific, Waltham, MA, USA). For each sample as well as reference RNA (Human XpressRef Universal Total RNA, QIAGEN, Hilden, Germany), the reaction was performed in a total volume of 20 µL and the RNA was diluted in nuclease-free water to an overall quantity of 500 ng per reaction. The reverse transcription reactions were performed using a Bio-Rad MJ Mini Personal Thermal Cycler (Bio-Rad, Hercules, CA, USA) with the following thermal conditions: 25 °C for 10 min, 37 °C for 120 min, and 85 °C for 5 min. The cDNA thus prepared was stored at −20 °C.

### 2.3. Targeted RNA Sequencing of Cancer Transcriptome Genes

Analyses were performed on a subset of 12 liver metastatic and 3 non-tumor control samples selected from total of 27 liver metastases and 31 control samples available. The 12 metastasis samples were selected based on a combination of biological relevance and technical feasibility. The primary objective was to identify DEGs between colorectal cancer liver metastases and adjacent non-tumor tissues. A secondary objective was to assess gene expression differences between *KRAS*-mutant and *KRAS*-wild-type metastases. To achieve these aims, samples were prioritized according to the following criteria: (i) high RNA quality, defined as an RNA Integrity Number (RIN) > 7; (ii) a sufficient RNA concentration for successful library preparation; (iii) a verified *KRAS* mutational status; and (iv) a balanced representation of both *KRAS*-mutant and *KRAS*-wild-type samples to allow for contrast. The gene panel supports robust DEG analysis even in moderately sized cohorts [18]. The differential gene expression (DGE) of 395 genes involved in 9 pathways (angiogenesis, apoptosis, cell cycle, senescence, DNA damage and repair, EMT, hypoxia, metabolism, and telomere and telomerase) was evaluated using next-generation RNA sequencing. The list of genes is provided in Supplementary Material S1. RNA-seq libraries were constructed using the commercially available targeted panel QIAseq Targeted RNA Human Cancer Transcriptome Panel (QIAGEN, Hilden, Germany).

The input RNA concentration for cDNA synthesis was 200 ng. Library construction was performed according to the manufacturer’s instructions, except for the second PCR, where we modified the number of cycles from 20 to 14. Each library incorporated a 12 bp unique molecular identifier (UMI) to collapse PCR duplicates to unique molecules, ensuring that the counts reflected unique transcript molecules and minimizing amplification bias [19]. The concentration of the libraries was measured using a QUBIT 3.0 fluorometer, and quality control was performed using an Agilent Bioanalyzer 2100. The 12 samples of liver metastases were sequenced in two runs on the MiSeq platform (Illumina, San Diego, CA, USA), using single-end 1× 150 bp reads. We obtained a mean of ~2.5 million reads per sample. For a 395-gene panel, this corresponds to >6000 reads per gene on average, which falls in the moderate-to-high coverage range (2000–15,000 reads per gene) recommended by the manufacturer. The list of samples with their clinicopathological characteristics, along with additional data, is provided in Supplementary Table S1. Subsequently, the metastasis group was divided based on the mutational status of the *KRAS* gene into *KRAS*-mutated (*KRAS*+) and *KRAS*-non-mutated (*KRAS*−) groups, which were compared with the controls as well as with each other.

### 2.4. Validation of RNA-Seq Data Using qPCR Method

The validation using qPCR was performed on the full patient cohort, comprising a total of 142 samples from CRC patients: 84 primary tumors, 27 liver metastases, and 31 non-tumor control tissues. Based on the DGE results from RNA sequencing, six genes from the EMT and hypoxia pathways were selected for qPCR validation: *SLC16A3*, *ANXA2*, *P4HA1*, *SPP1*, *KRT19*, and *LGALS3*. This validation was performed using TaqMan probes (Applied Biosystems) for each of the target genes—Hs00358829_m1—FAM-MGB (*SLC16A3*); Hs00914594_m1—FAM-MGB (*P4HA1*); Hs00733396_m1—FAM-MGB (*ANXA2*); Hs00959010_m1—VIC-MGB (*SPP1*); Hs00173587_m1—VIC-MGB (*LGALS3*)—and a custom probe for *KRT19* (FAM-MGB) designed to hybridize at the exon 3–4 junction (5′ GGAAGAGCTGGCCTACCTGAAGAAGAACCATGAGGAGGAAATCAGTACGCTGAGGGGCCAAGTGGGAGGCCAGGTCAGTGTG 3′), as well as a probe for the housekeeping gene actin beta (*ACTB*), Hs99999903_m1—VIC-MGB. Each duplex reaction was carried out in a total volume of 10 µL consisting of 5 µL of TaqMan Fast Advanced Master Mix (Thermo Fisher Scientific, MA, USA), 0.5 µL of FAM-labeled TaqMan probe, 0.5 µL of VIC-labeled TaqMan probe, 1 µL of cDNA, and 3 µL of nuclease-free water. The FAM and VIC assays were paired for duplex reactions as follows: *SLC16A3* + *ACTB*; *ANXA2* + *ACTB*; *P4HA1* + *SPP1*; *KRT19* + *LGALS3*. Each duplex reaction was analyzed in duplicate.

The thermal cycling conditions for the qPCR were as follows: incubation at 50 °C for 2 min, the activation of polymerase at 95 °C for 20 s, and 40 cycles of denaturation at 95 °C for 3 s and annealing/extension at 60 °C for 30 s. All the qPCR experiments were conducted on a 7500 Fast Real-Time PCR System (Applied Biosystems, Waltham, MA, USA) employing MicroAmp™ Fast Optical 96-well reaction plates (0.1 mL volume, Applied Biosystems, Waltham, MA, USA).

### 2.5. Data and Statistical Analysis

Data analysis was conducted using the BaseSpace Sequence Hub platform (Illumina, San Diego, CA, USA; https://euc1.sh.basespace.illumina.com, accessed on 1 April 2025), employing the DRAGEN RNA (version 4.3.6) and DRAGEN Differential Expression (version 4.3.6) applications for the secondary analysis of RNA transcripts. Initially, FASTQ files were imported into the DRAGEN RNA pipeline, which performed read mapping, transcript alignment to the reference transcriptome, and the quantification of gene expression via read counting and normalization. The alignment was carried out using Illumina’s proprietary spliced aligner against the GRCh38 human reference genome and the GENCODE v32 annotation. The transcript annotation was normalized using TPM (transcripts per kilobase million), which accounts for gene length and sequencing depth, enabling the inter-sample comparison of expression levels. Subsequently, the DRAGEN Differential Expression tool (v4.3.6) was used to identify differentially expressed genes (DEGs), applying the DESeq2 algorithm to quantify the expression of each gene within the panel for every sample, enabling the comparison of expression between groups. Genes were considered differentially expressed if they had an adjusted *p*-value (padj) < 0.05, controlling the False Discovery Rate (FDR). Heatmaps of DEGs were automatically generated using rlog-transformed count data (regularized log transformation form DESeq2), with the expression values standardized to Z-scores for each gene individually. All the analyses were performed using the default settings for the respective tools. Downstream analyses were restricted to significantly differentially expressed genes belonging to hypoxia- and EMT-related pathways, with priority given to those showing the strongest differential expression (padj < 0.05 and log2FC > 2), in line with the primary aim of the study.

The fold change (FC) was calculated for each of the 6 validated genes using the ΔΔct method. The relative change in expression between groups was evaluated based on the comparisons, according to the sample type (tumor, primary tumor, liver metastasis, control sample); different clinical parameters (clinical stage, TIL and PTIL status); and mutational status of the *KRAS*, *PIK3CA*, *SMAD4*, *APC*, and *TP53* genes (+mutated; −non-mutated).

A list of all the criteria is provided in Table 2. After calculating the FC, this value was logarithmically transformed (log_2_FC) and subjected to a statistical analysis using Student’s *t*-test with Welch’s correction. A gene was considered differentially expressed if the absolute value of log_2_FC > 0.5 and *p* < 0.05. All analyses were performed using Microsoft Excel, and the raw data is available upon request. The boxplots were made with ggplot2 (v3.5.1) in R (v4.4.1; R Foundation for Statistical Computing, Vienna, Austria). Statistical significance was measured with Student’s *t*-tests in R and is indicated by asterisks: * *p* < 0.05, ** *p* < 0.01, ** *p* < 0.001 (https://github.com/Emile-Jn/SPP1-CRC-boxplots, accessed on 24 July 2025, Supplementary Material S4).

### 2.6. Public Data Survival Analysis

To evaluate the prognostic relevance of selected genes, we used the publicly available GEPIA2 web tool (http://gepia2.cancer-pku.cn/, accessed on 20 July 2025), which provides access to The Cancer Genome Atlas (TCGA) datasets, and Kaplan–Meier survival analysis was performed using the COAD dataset (colorectal adenocarcinoma), focusing on overall survival. The analysis was conducted separately for *SPP1* and *P4HA1*, using median expression as the cutoff. The hazard ratios (HRs), log-rank *p*-values, and sample sizes were recorded.

## 3. Results

### 3.1. RNA Sequencing

The DGE of 395 genes of the cancer transcriptome was evaluated in 12 liver metastasis samples and compared to control non-tumor samples (n = 3). The metastatic samples were subsequently divided based on the *KRAS* gene mutation status, into a *KRAS*+ group (n = 5) and *KRAS*− group (n = 7), which were also compared to each other. A total of four comparisons were performed: metastases vs. control, *KRAS*+ metastases vs. control, *KRAS*− metastases vs. control, and *KRAS*+ metastases vs. KRAS− metastases.

**Figure 1:**
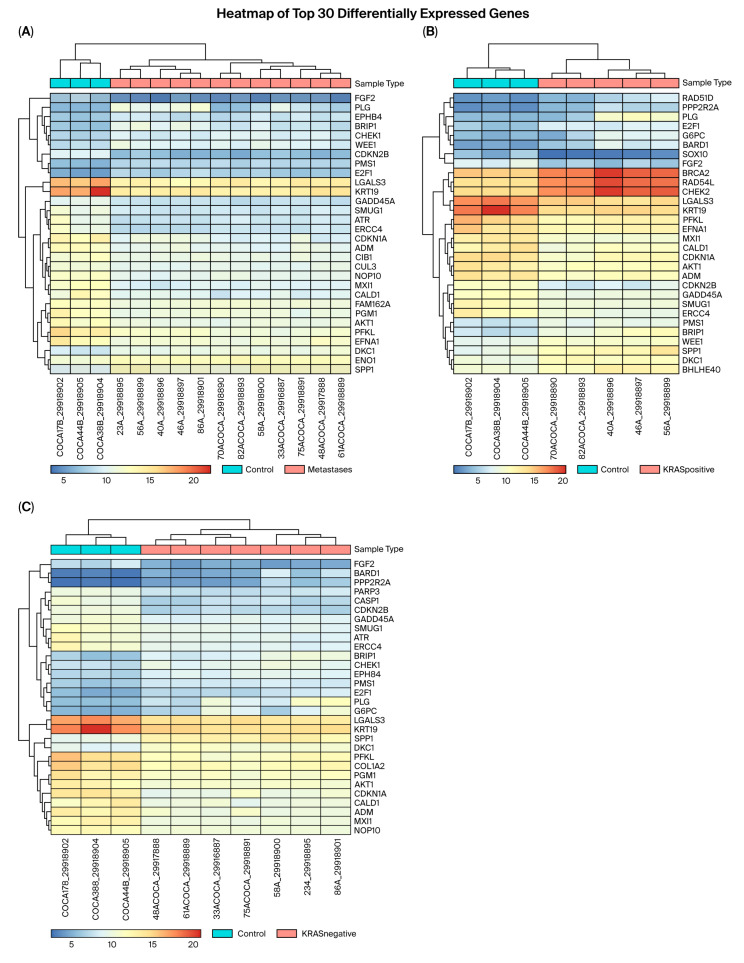
Heatmaps of top 30 DEGs. The vertical axis represents the names of the DEGs. The horizontal axis represents the samples (ending with **B**—non-tumor control samples; ending with **A**—liver metastasis samples). The red color represents upregulation, while blue represents downregulation. (**A**): A heatmap of the top 30 DEGs when comparing all the metastasis samples with non-tumor samples. (**B**): A heatmap of the top 30 DEGs when comparing the *KRAS*+ metastases with non-tumor samples. (**C**): A heatmap of the top 30 DEGs when comparing the *KRAS*− metastases with non-tumor samples.

The most DEGs, up to 195, were identified when comparing metastases and control non-tumor samples. This was followed by 162 genes that were differentially expressed between *KRAS*− metastases and controls, and 136 genes between *KRAS*+ metastases and controls. The comparison of *KRAS*+ and *KRAS*− metastases revealed a differential expression of only a single gene, specifically *CASP1*. Heatmaps of the top 30 DEGs were created for the control vs. metastases, control vs. KRAS+ metastases, and control vs. *KRAS−* metastases comparisons (Figure 1A–C). Each heatmap represented genes from all nine signaling pathways; however, the most abundantly represented pathway in all the comparisons was the cell cycle pathway. All the results regarding DEGs from all the comparisons are provided in Supplementary Table S2.

For further analysis, we focused on hypoxia- and EMT-related genes. When comparing all metastases versus controls, five hypoxia genes (*LGALS3*, *ATR*, *ADM*, *MXI1*, and *FAM162A*) and three EMT genes (*KRT19*, *CALD1*, and *SPP1*) were among the top 30 DEGs (*p* < 0.001). Except for *SPP1*, all these genes exhibited lower expression levels in metastases compared to control non-tumor samples. *KRT19* showed the most pronounced reduction in expression (log_2_FC = −6.71), while *LGALS3* had the third most reduced expression (log_2_FC = −4.75), with their expression levels in metastases being less than 1% and 4% of the control, respectively (*p* < 0.001). Conversely, *SPP1* expression was the third most increased in metastases compared to control samples, showing over 65-fold upregulation (log_2_FC = 6.029) (*p* < 0.001).

Dividing the metastases based on the mutational status of the KRAS gene, and subsequently comparing KRAS+ metastases with controls, showed that four hypoxia genes (*LGALS3*, *MXI1*, *ADM*, and *BHLHE40*) and four EMT genes (*SOX10*, *KRT19*, *CALD1*, and *SPP1*) were among the top 30 DEGs. Apart from the *SPP1* and *BHLHE40* genes, all the hypoxia- and EMT-related genes were downregulated in the metastases (*p* < 0.001). Specifically, the *KRT19*, *SOX10*, and *MXI1* genes exhibited the most significant downregulation in *KRAS*+ metastases relative to control non-tumor samples, with log_2_FC values of −7.28, −6.33, and −4.73, respectively. Conversely, the expression of the *SPP1* gene in the *KRAS*+ metastases was over 43-fold higher than that in the control samples (log_2_FC = 5.44) (*p* < 0.001).

In the analysis of *KRAS*− metastases versus non-tumor samples, the genes identified included *ATR*, *LGALS3*, *ADM*, and *MXI1* from the hypoxia pathway, and *CALD1*, *COL1A2*, *KRT19*, and *SPP1* from EMT. Consistent with observations in all metastases, overexpression was observed only for the *SPP1* gene, which exhibited an almost 83-fold upregulation compared to the controls (log_2_FC = 6.37) (*p* < 0.001). Conversely, the *KRT19* gene was again found to be the most downregulated in *KRAS*− metastases, showing a reduction of nearly 99% (log_2_FC = −6.36) (*p* < 0.001).

We also considered the differential expression of EMT and hypoxia gene transcripts across all comparisons. Notably, no differentially expressed EMT or hypoxia transcripts were identified when comparing *KRAS*− and *KRAS*+ metastases. Based on the results for both differentially expressed genes and transcripts, six key genes were prioritized for DGE validation: *SLC16A3*, *ANXA2*, and *P4HA1* were selected based on the differential expression of their transcripts, while *SPP1*, *KRT19*, and *LGALS3* were chosen from the top 30 DEGs. The differentially expressed transcripts of EMT and hypoxia genes for all comparisons (control vs. metastases, control vs. KRAS+ metastases, and control vs. KRAS− metastases) are detailed in Supplementary Table S3.

### 3.2. Validation of Differential Gene Expression

To confirm the RNA sequencing results, the DGE of six selected genes—*SLC16A3*, *ANXA2*, *P4HA1*, *SPP1*, *LGALS3*, and *KRT19*—involved in the hypoxia and EMT pathways was assessed using qPCR. The results were validated in a larger cohort comprising primary tumor tissues, liver metastasis samples, and control non-tumor samples.

**Figure 2:**
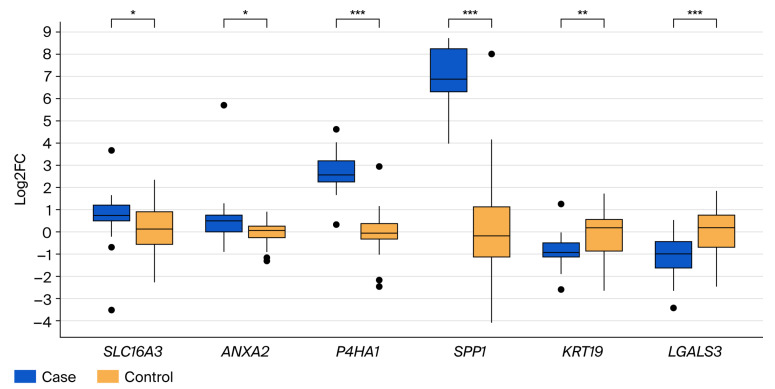
Boxplots showing fold change differences for SLC16A3, *ANXA2*, *P4HA1*, *SPP1*, *KRT19*, and *LGALS3* genes in liver metastases (case) compared to non-tumor (control) tissues (* *p* < 0.05; ** *p* < 0.01; *** *p* < 0.001). **·** outlier.

The differential gene expression analysis comparing primary colorectal tumor tissues and liver metastases to non-tumor control tissues identified significant expression changes in all six validated genes (Supplementary Table S4). Specially, the genes *SLC16A3*, *ANXA2*, *P4HA1*, and *SPP1* were significantly upregulated, while *KRT19* and *LGALS3* were significantly downregulated in tumor tissues (*p* < 0.05). When focusing exclusively on liver metastases compared to control tissues (Figure 2), *P4HA1* and *SPP1* displayed the highest and most significant increase in expression (*p* < 0.001). *SLC16A3* and *ANXA2* exhibited moderate yet significant increases, whereas *KRT19* and *LGALS3* showed significant reductions in expression (*p* < 0.05).

**Figure 3:**
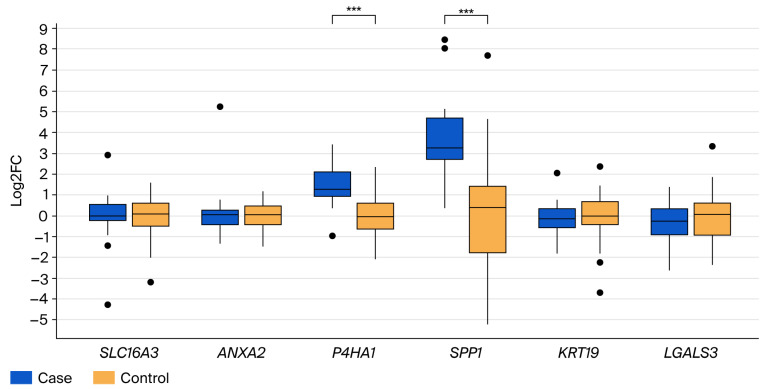
Boxplots showing fold change differences for *SLC16A3*, *ANXA2*, *P4HA1*, *SPP1*, *KRT19*, and *LGALS3* genes in liver metastases (case) compared to primary tumor (control) samples (*** *p* < 0.001). **·** outlier.

A direct comparison of liver metastases with primary tumor tissues (Figure 3) highlighted significantly elevated expression of *P4HA1* and *SPP* in the metastatic samples (*p* < 0.001). The expression changes for *SLC16A3*, *ANXA2*, *LGALS3*, and *KRT19* between primary tumors and metastases were minor and did not reach statistical significance.

**Figure 4:**
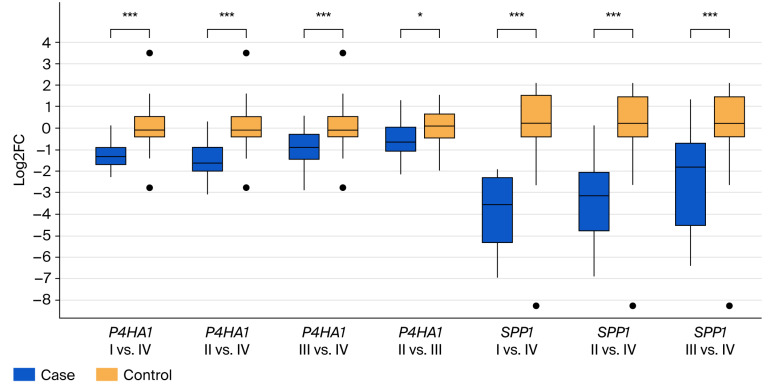
Boxplots comparing the changes in expression of *P4HA1* and *SPP1* genes between distinct CRC stages. The specific pairwise comparisons between CRC stages are indicated within the figure (* *p* < 0.05, *** *p* < 0.001). **·** outlier.

Comparative analysis between distinct CRC stages (Figure 4) revealed significant differences in the expression levels of *P4HA1* and *SPP1*, particularly evident between stage IV tumors and earlier stages (stages I–III; *p* < 0.001). Unlike that of the *SPP1* gene, the expression of *P4HA1* was significantly upregulated in stage III compared to II (*p* < 0.05). Concerning the *SPP1* gene, a trend of increasing expression from the lowest in stage I, to higher in stages II and III, to the highest in stage IV of the disease was observed. Such a clear trend was not observed for the *P4HA1* gene.

**Figure 5:**
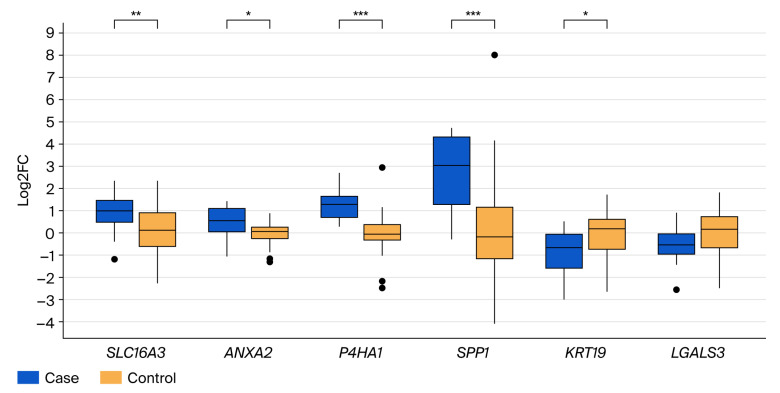
Boxplots showing fold change differences for *SLC16A3*, *ANXA2*, *P4HA1*, *SPP1*, *KRT19*, and *LGALS3* genes in stage I samples (case) compared to control non-tumor (control) samples. The log_2_FC of the control samples represents a value close to 0 (* *p* < 0.05; ** *p* < 0.01; *** *p* < 0.001). **·** outlier.

For the *SLC16A3*, *ANXA2*, *KRT19*, and *LGALS3* genes, we did not observe a significant difference in expression between individual clinical stages (Supplementary Table S4). On the contrary, gene expression analysis in early-stage CRC (stage I) versus controls (Figure 5) showed significant differences across all the studied genes, with the exception of *LGAL3.* The genes *SLC16A3*, *ANXA2*, *P4HA1*, and *SPP1* were significantly upregulated, while the gene *KRT19* was significantly downregulated, in stage I compared to adjacent non-tumor samples (*p* < 0.05). In this comparison, the genes *P4HA1* and *SPP1* again presented with pronounced upregulation. Consistently, these genes were also significantly upregulated in stage IV relative to the combined stages I + II + III (*p* < 0.001). Furthermore, altered expression based on the TIL and PTIL status of the samples was observed for only a single gene, *SLC16A3* (*p* < 0.05). Its expression was slightly upregulated in TIL-positive compared to TIL-negative samples. Moreover, *SLC16A3* was the only gene slightly more highly expressed when comparing primary tumors to controls than when comparing metastases to controls (Supplementary Table S4).

**Figure 6:**
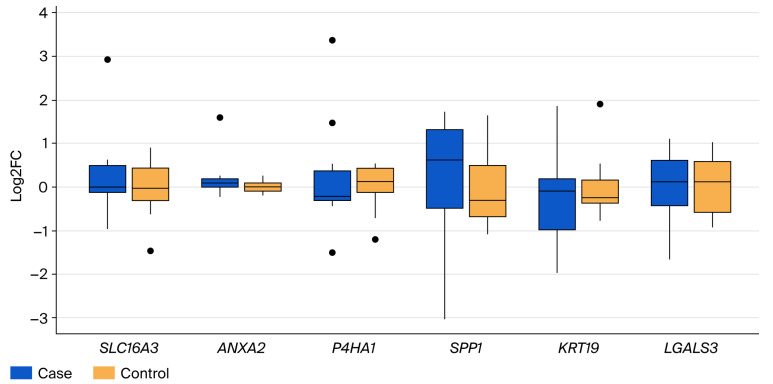
Boxplots showing fold change differences for *SLC16A3*, *ANXA2*, *P4HA1*, *SPP1*, *KRT19*, and *LGALS3* genes in *KRAS+* metastases compared to *KRAS*− metastases. The log_2_FC of the *KRAS*− metastatic samples represents a value close to 0. **·** outlier.

In our study, we also focused on how the mutational status of driver genes, mainly *KRAS*, but also others such as *PIK3CA*, *APC*, *SMAD4*, and *TP53*, influenced the expression of selected genes. To explore the effect of mutations in these genes, we performed several comparisons: tumors positive vs. negative for the mutation, tumors positive for the mutation vs. tumors with zero mutations in driver genes, and tumors with mutations in more than three driver genes vs. tumors with zero mutations and vs. tumors with fewer than three mutations in driver genes. For the *KRAS* gene, we also compared the expression between *KRAS*+ and *KRAS*− liver metastases. Based on the mutational status of the *KRAS* gene, we did not observe any significant change in the expression of any of the selected genes, neither in all the *KRAS*+ tumors nor in *KRAS*+ metastases. An increase in expression was observed for *SLC16A3*, *ANXA2*, and *P4HA1*; a decrease for *KRT19*; and a log_2_FC value very close to 0 for *SPP1* and *LGALS3* (Figure 6). The expression of validated genes was also compared between MSI and MSS CRC tissues, but without statistically significant results.

Regarding mutations in other genes, significantly reduced expression of *SLC16A3* was observed in *TP53*+ tumors compared to both *TP53*− tumors and those with zero mutations in driver genes (*p* < 0.01). On the contrary, the *ANXA2* gene was significantly overexpressed in *PIK3CA*+ tumors compared to tumors with zero mutations in driver genes (*p* < 0.05). Furthermore, the *ANXA2* gene was also significantly overexpressed in tumors with three or more mutations in driver genes compared to tumors with zero mutations and tumors with fewer than three mutations in driver genes (*p* < 0.05). The expression of the *SLC16A3* and *ANXA2* genes was not affected by mutations in other genes. Our results also suggest only non-significant changes in the expression of *P4HA1*, *SPP1*, *KRT19*, and *LGALS3* based on the mutational status of driver genes. A comprehensive list of all the comparisons and their respective *p*-values is provided in Supplementary Table S4. To address the potential impact of inter-individual variability, we performed a separate analysis on a subset of matched primary tumors and adjacent non-tumor samples from the same patients. This paired comparison confirmed the significant differential expression of all the validated genes, supporting the robustness of our original findings (Supplementary Table S5).

### 3.3. Survival Association of SPP1 and P4HA1 in Public Colorectal Cancer Datasets

To explore the prognostic significance of SPP1 and P4HA1, we analyzed overall survival in the TCGA COAD dataset using the GEPIA2 platform [20]. Patients were stratified into high- and low-expression groups based on median expression levels. For SPP1, high expression was associated with a hazard ratio (HR) of 1.5 compared to low expression, although the difference did not reach statistical significance (log-rank *p* = 0.13; n = 135 in each group). Similarly, P4HA1 expression was also associated with an HR of 1.5 (log-rank *p* = 0.098; n = 135 per group). While not statistically significant, both trends suggest that increased expression of these genes may be linked to poorer overall survival in colorectal cancer (Supplementary Materials S2 and S3). Notably, in publicly available datasets, SPP1 expression was not consistently associated with overall survival, suggesting that, while expression varies with disease stage, outcome prediction may depend on additional clinical or molecular factors. Because GEPIA2 provides univariate survival curves without covariate adjustment [20], these results should be interpreted as exploratory, hypothesis-generating evidence rather than proof of independent prognostic value.

## 4. Discussion

DGE analysis is currently a widely used method that significantly contributes to characterizing the molecular features of tumors, including CRC. Transcriptome and DGE analysis using RNA sequencing has also proven effective in establishing the classification system for CRC [21]. RNA sequencing and subsequent analyses provided us with results regarding DGE between metastatic samples (all metastases, *KRAS+* metastases, *KRAS−* metastases) and control samples. In our study, we subsequently focused on DGE in metastatic CRC, with a specific emphasis on identifying and validating the DGE of six EMT- and hypoxia-related genes: *SLC16A3*, *ANXA2*, *P4HA1*, *SPP1*, *KRT19*, and *LGALS3*. For each of the six genes, we performed comparisons based on sample type, clinical parameters, and the mutational status of driver genes. However, for some genes, we observed a slight discrepancy between the qPCR and RNA sequencing results. Several studies have noted that the correlation between RNA sequencing and qPCR data varies, being higher for some genes and lower for others [22,23]. In our case, this was evident with genes such as *KRT19* and *LGALS3*. While RNA sequencing identified them as among the most DEGs, qPCR did not confirm such a pronounced decrease in expression. Our findings underscore the critical need for validating RNA sequencing outputs using complementary methods, such as qPCR, to guarantee reliable and relevant results.

A limitation of our study is the modest size of the RNA-seq discovery cohort (12 liver metastases and 3 controls). Power simulations and empirical benchmarks indicate that two to three replicates per group generally only enable the detection of the strongest changes, whereas five to six replicates are typically needed for the reliable detection of moderate-fold changes [24,25]. To address this constraint, we used a 395-gene panel rather than whole transcriptome RNA-seq, focusing our sequencing on cancer-relevant genes and transcripts. This design yielded ~2.5 million reads per sample, corresponding to >6000 reads per gene on average. In addition, the UMI-based deduplication minimized PCR noise [18], and restricting analysis to 395 genes reduced the multiple-testing burden compared with whole transcriptome designs [18,26]. By combining a targeted panel approach with a pathway-focused strategy, we were able to mitigate some of the limitations of the small discovery cohort and increase the significance of the findings. In line with our aim, we further focused the analysis on genes showing a log2 FC >2 and involved in EMT and hypoxia pathways. Most importantly, all the candidate genes were validated in a substantially larger cohort by qPCR. This two-step design—a small discovery cohort followed by a larger validation study—strengthens the evidence and is consistent with the recommendations that discoveries from modest discovery cohorts be confirmed in larger sets [25].

RNA sequencing revealed increased expression of the genes *SLC16A3*, *ANXA2*, *P4HA1*, and *SPP1* in liver metastasis samples compared to control samples. In contrast, the genes *KRT19* and *LGALS3* were significantly downregulated in the metastases. The RNA sequencing results suggest that the expression of transcript ENST00000619321.1 of the *SLC16A3* gene was significantly increased in the metastases, in both *KRAS+* and *KRAS−*, compared to non-tumor tissues. These findings are consistent with those of Li et al. (2021), who observed increased expression of *SLC16A3* in several tumor types, as well as with the results of Wang et al. (2022), who even observed a trend of increasing *SLC16A3* expression from the lowest in healthy tissues, through primary tumors, to the highest in metastases [27,28]. Our results do not indicate this trend, as *SLC16A3* expression was slightly higher in primary CRC samples than in metastases when compared to control samples, and the comparison between primary tumors and metastases did not show a significant change in the expression of this gene. Regarding the role of *SLC16A3* as a prognostic marker, several research groups have demonstrated that high expression of this gene is associated with poor prognosis in different types of cancer [27,28,29]. Xue and colleagues (2021) observed a significant increase in the expression of this gene across stages I–IV of lung adenocarcinoma compared to control samples [30]. To our knowledge, however, results for CRC are not available. In our comparative analysis of expression between different clinical stages of the disease, we observed only a negligible and non-significant change in the expression of this gene.

We obtained similar results in the case of the *ANXA2* gene, which was selected for validation based on the RNA sequencing results, where its transcript ENST00000421017.6 was among the most differentially expressed. Several studies suggest that *ANXA2* is an important player in carcinogenesis, with increased expression observed in various tumor types, including CRC [31,32]. Similarly, in our case, we observed significantly increased expression in metastases compared to control samples. The results of Rocha et al. further suggest that *ANXA2* expression in CRC is significantly elevated in stage IV compared to stages I–III [31]. In our comparison across clinical stages, we did observe higher expression in stage IV than in stages I–III, but this difference was not statistically significant. However, we did observe a significant increase in expression in stage I samples compared to controls, which may indicate a role for *ANXA2* even in the early phases of tumorigenesis. For the *ANXA2* gene, its potential clinical relevance has been proposed in relation to CRC, as well as prostate and breast cancer [33,34,35]. In CRC, increased expression has been associated with poorly differentiated tumors, advanced stage, and lymph node positivity [28]. Moreover, it has been shown that *ANXA2* expression varies depending on the specific CMS subtype of the tumor, with the highest expression in CMS1, followed by CMS3 and CMS4, and the lowest in CMS2 [31]. Although CMS1 and CMS3 consist of tumors with the MSI phenotype, we did not observe a significant difference in *ANXA2* expression between MSI and MSS tumors. However, our results suggest that increased *ANXA2* expression may be associated with the occurrence of metastases as well as early-stage CRC, indicating its potential as a biomarker, though further studies are needed to confirm this.

Regarding the *P4HA1* gene, DGE analysis revealed two transcripts—ENST00000307116.6 and ENST00000373008.6—to be differentially expressed when comparing control samples with all metastases and with *KRAS−* metastases. In the case of *KRAS+* metastases, only the latter transcript showed differential expression. Increased expression levels in tumor tissue have been observed in several cancer types, including breast cancer, esophageal carcinoma, and lung and colorectal adenocarcinomas [36]. The role of *P4HA1* in CRC carcinogenesis is further supported by findings that silencing its expression in CRC cell lines led to reduced proliferation, invasion, and migration, as well as the inhibition of tumor growth—though not of metastasis [37]. Several groups have observed that *P4HA1* expression is elevated in both primary tumors and metastases compared to normal tissue in CRC [36,37]. Our experiments on a CRC tissue cohort showed that *P4HA1* expression was significantly increased in both primary tumors and metastases compared to non-tumor samples, with a significant difference between primary tumors and metastases also observed. Additionally, expression comparisons across CRC clinical stages revealed an increase in stage IV compared to stages I, II, and III, as well as in stage I samples compared to non-tumor tissues, suggesting a role for *P4HA1* in the early stages of tumorigenesis. These results are consistent with those of Agarwal et al., who observed a significant increase in *P4HA1* expression across CRC stages I–IV compared to normal intestinal tissue [37]. Tanaka et al. correlated *P4HA1* expression with clinicopathological data, demonstrating that high *P4HA1* expression correlates with poor tumor differentiation, right-sided localization, and loss of *MMR* gene function [38]. In the case of MSI tumors, whose development is closely linked to *MMR* gene dysfunction, we observed only an insignificant change in *P4HA1* expression compared to MSS tumors. The role of *P4HA1* as a prognostic marker has been proposed by several research groups. For example, Tanaka et al. stated that high P4HA1 protein expression appears to be a poor prognostic marker for patients in the early stages of CRC [38]. Based on a meta-analysis of genomic and proteomic data, a panel of four proteins, including P4HA1, has been proposed as potential plasma biomarkers for early CRC diagnosis [39]. It has also been observed that CRC patients with lower *P4HA1* expression have higher survival rates than those with higher expression [40]. Our results support the role of *P4HA1* as a candidate biomarker, though further studies are needed to confirm this.

The *SPP1* gene, which influences tumor growth, adhesion, and invasion in pathological processes [41], ranked among the top 30 most DEGs in all three comparisons and showed significant overexpression in metastatic tissues. Among all six validated genes, *SPP1* exhibited the greatest changes in expression, particularly in metastases, but also in primary tumors compared to non-tumor samples. When we examined expression across individual clinical stages, we observed a gradual trend of increasing expression—from the lowest in stage I, to higher in stages II and III, and to the highest in stage IV. We also observed a significant difference in expression between stage IV and the other stages. Our results suggest that, based on expression levels, it may be possible to distinguish CRC stages I–III from stage IV, as well as from metastases, where expression is markedly elevated. A similar trend of increasing expression from stage I to stage IV in CRC was observed by Jiang et al. [41], even in tissue, plasma, and serum samples. It was shown that *SPP1* is significantly overexpressed in breast cancer, and colorectal and lung adenocarcinomas, as well as in other tumor types [42]. The authors also reviewed publications on *SPP1* expression in CRC and concluded that none of them reported decreased expression of this gene in CRC [42]. We observed significantly increased expression of *SPP1* in stage I of the disease compared to control samples, which may indicate that *SPP1* plays a role not only in advanced stages but also in the early phases of carcinogenesis. For *SPP1*, increased expression in rectal and colon cancer has been identified as an unfavorable factor for progression-free and overall survival [43]. Furthermore, multiple research groups have proposed osteopontin as a prognostic marker in patients with resectable liver metastases from CRC [42,43]. Another study mentioned *SPP1* in the context of a potential therapeutic target—specifically, *SPP1*-positive macrophages that, along with others, promote CRC metastasis and could be targeted in future treatments for liver metastases [44,45]. The therapeutic relevance of SPP1 in the context of metastasis as supported by the previous report [45] was not experimentally addressed in this study. Our data extend prior observations by highlighting differential expression in liver metastases, suggesting its potential to provide therapeutic leads for future studies. Our results indicate that *SPP1* is relevant in both early and advanced stages of CRC, especially in metastases, and that its expression increases proportionally with disease progression. Our findings, along with other studies, suggest that *SPP1* is an important player in CRC carcinogenesis, though its role as a potential biomarker remains open.

Recent studies suggest that the upregulation of *SPP1* may promote malignant progression and therapeutic resistance via activation of the KRAS/MEK signaling pathway, leading to a poor prognosis [46]. Although this mechanism has been primarily studied in cancers other than colorectal cancer, the biological rationale linking *SPP1* overexpression to KRAS-driven signaling and resistance to anti-EGFR therapies such as cetuximab makes it a relevant and compelling area for further investigations in CRC. In our current cohort, however, we did not observe a significant correlation between SPP1 expression levels and the presence of *KRAS* mutations. Nevertheless, given the high prevalence of *KRAS* mutations and frequent resistance to targeted therapies in CRC, future functional studies exploring potential interactions between *SPP1* and KRAS/MEK signaling remain warranted. Such research could yield critical insights into the prognostic relevance and therapeutic targeting of SPP1 in colorectal cancer. Moreover, the Hippo signaling pathway has recently emerged as a key regulator of tumor progression, EMT, and therapy resistance in colorectal cancer, particularly through its interaction with *KRAS*-driven oncogenic signaling [47,48]. Given the high prevalence of *KRAS* mutations in CRC [49] and growing evidence that Hippo pathway activity contributes to the resistance mechanisms in *KRAS*-mutant CRC, future analyses in out cohort will aim to evaluate gene expression in relation to *KRAS* mutational status, Hippo signaling pathway components, and their associated transcriptional targets and modulators. Investigating these relationships in the context of metastatic progression may help uncover novel biomarker candidates and provide mechanistic insights into therapy resistance in advanced CRC.

To assess the prognostic potential of *SPP1* and *P4HA1*, we performed a survival analysis using publicly available colorectal cancer data from the TCGA COAD dataset via GEPIA2. Patients with high expression of both genes showed a trend toward poorer overall survival compared to those with low expression. Specifically, high *SPP1* expression was associated with a hazard ratio (HR) of 1.5 (*p* = 0.13), and high *P4HA1* expression also showed an HR of 1.5 (*p* = 0.098), with n = 135 in both expression groups. While our cohort demonstrated statistically significant associations, the public data did not reach significance; however, the direction of the effect was consistent with our findings and supports the potential of these genes as candidate prognostic markers in colorectal cancer. The discrepancy in statistical significance highlights the context-dependence of *SPP1* expression and underscores the need for further survival analysis in metastasis-specific datasets. Moreover, the prognostic analysis based on TCGA data did not adjust for clinical covariates such as age, stage, or molecular subtypes. Because GEPIA2 provides univariate survival curves without covariate adjustment [20], these findings should be interpreted as exploratory. Establishing whether *SPP1* or *P4HA1* is an independent prognostic biomarker will require validation in larger, clinically annotated datasets using multivariate Cox regression models that account for age and other established prognostic factors.

The other two genes related to EMT and hypoxia that ranked among the top 30 most DEGs and were selected for validation—*KRT19* and *LGALS3*—showed significantly reduced expression in metastases. Among all the genes, *KRT19* exhibited the most pronounced downregulation across all three comparisons. Although the validation experiments did not confirm such a strong reduction in expression, we still observed differential expression of *KRT19* between primary tumors and metastases compared to non-tumor samples, though with only minimal differences between the former two groups. Several studies suggest that *KRT19* expression is elevated in CRC, as well as in breast and lung cancers, compared to control samples [50,51]. On the other hand, reduced *KRT19* expression has been observed in cutaneous melanoma, acute lymphoblastic leukemia, and adrenocortical carcinoma [51]. Saha et al. also found that *KRT19* knockdown in CRC led to the suppression of WNT/Notch signaling and subsequent inhibition of cancer progression [50]. Interestingly, the same authors studied the effect of *KRT19* silencing in breast cancer and observed the suppression of WNT but enhancement of Notch signaling, which led to increased tumorigenic properties. This indicates that *KRT19* may play different roles in carcinogenesis depending on how it modulates signaling pathways [50]. A similar trend, where *KRT19* knockdown led to the increased proliferation, migration, invasion, and survival of breast cancer cells, was observed by Ju et al., who also confirmed that silencing *KRT19* resulted in increased tumor formation in xenograft models [52]. Our results suggest a similar pattern, as we observed a significant decrease in *KRT19* expression in stage I samples compared to non-tumor tissues, indicating a potential role for *KRT19* in the early stages of CRC carcinogenesis. Interestingly, in thyroid cancer, increased *KRT19* expression was observed only in tumors with the *BRAF V600E* mutation, and *BRAF* overexpression was shown to induce *KRT19* expression [53]. If this also applies to CRC, it could explain the low expression levels of *KRT19* in our cohort, where the frequency of *BRAF* mutation was less than 6% in primary tumors and 0% in metastases. The expression profile of *KRT19* appears to be quite complex and influenced by many factors. The same applies to downstream signaling pathways, which may be regulated differently across tumor types, often leading to contradictory results. Publications on KRT19 reveal its role as a potential predictive biomarker and therapeutic target, for example, in pancreatic cancer, as well as a possible therapeutic target for TGF-β receptor inhibition [54,55]. Others have proposed its potential as a prognostic marker in lung cancer [56]. Because our results indicate a significant decrease in *KRT19* expression in tumor samples, which contradicts other findings related to CRC, and because *KRT19* appears to be a promising biomarker, it is essential that further attention be given to it in the future.

Analysis of the *LGALS3* gene revealed results similar to those for *KRT19*, a marked decrease in expression in metastases based on RNA sequencing, which was also confirmed to a lesser extent by qPCR. However, *LGALS3* expression was slightly more reduced in metastases than in primary tumors when compared to non-tumor samples. A significant reduction in both the gene and protein expression of *LGALS3* in colorectal adenocarcinoma was also observed by Ren et al. [57]. In addition, decreased expression was also reported in breast, lung, and bladder cancers compared to control samples [57]. These findings are consistent with other studies suggesting that increased *LGALS3* protein expression induces lysosomal damage and subsequent lysophagy, thereby inhibiting CRC cell growth [58]. On the other hand, when comparing individuals with CRC to those with high-risk adenomas, the plasma levels of galectin-3 were higher in the CRC group, which may suggest an increasing trend in expression. Higher expression of this protein in CRC tissues compared to non-tumor tissue was also observed by Tao et al. [59]. The authors also correlated expression with clinicopathological characteristics and found that expression correlated with tumor size, tumor differentiation, and significantly lower median progression-free survival. As with *KRT19*, studies on *LGALS3* in CRC report both decreased and increased expression—whether at the gene or protein level. These discrepancies may be due to complex regulation influenced by many factors. It was suggested that galectin-3 plays a role as a marker of poor prognosis in CRC. This could be explained by one of its proposed functions: linking WNT and STAT3 signaling [59]. If confirmed, galectin-3 could potentially be used as a therapeutic target in the future [60]. Given its involvement in metastasis and proliferation, *LGALS3* appears to be an important player in carcinogenesis and should be a subject of future investigation.

One of our study’s aims was to identify DEGs between *KRAS+* and *KRAS−* metastatic CRC. Our RNA sequencing results showed that only caspase-1 was differentially expressed between these groups. Despite these findings, and after reviewing available literature, which revealed no direct studies on the relationship between the DGE of EMT- and hypoxia-related genes and *KRAS* mutational status, we proceeded to validate the expression of all six genes between *KRAS+* and *KRAS−* samples. Our results showed that none of the validated genes exhibited significantly altered expression between these samples, suggesting that the presence of the *KRAS* gene mutation does not affect the expression of any of the selected genes, in either all tumor samples or in metastases. These findings are consistent with publications on DGE in *KRAS+* and *KRAS−* CRC, which do not mention any of the validated genes in the context of DGE [61,62,63]. Regarding expression changes based on the mutational status of other genes (*PIK3CA*, *SMAD4*, *APC*, and *TP53*), we observed such changes only for *SLC16A3* and *ANXA2*. The expression of *SLC16A3* was significantly reduced in *TP53+* tumors compared to both *TP53−* and wild-type tumors. In contrast, results for lung adenocarcinoma suggest the opposite trend—an increase in *SLC16A3* expression in *TP53*-mutated tumors [30]. This inconsistency may be due to differences between colon and lung adenocarcinomas. However, to our knowledge, no such data are available for CRC. In both scenarios, further studies are needed to clarify the mechanism of *SLC16A3* expression regulation via TP53. We observed increased expression of *ANXA2* in *PIK3CA*+ tumors compared to wild-type tumors, as well as in tumors with three or more mutations compared to wild-type tumors and those with fewer than three mutations. Unfortunately, studies focusing on *ANXA2* expression in the context of *PIK3CA* mutations are, to our knowledge, not available. Therefore, we can only hypothesize whether the increased expression of *ANXA2* is a consequence of activating mutations in *PIK3CA* and the subsequent activation of signaling pathways that may influence the transcription of genes including *ANXA2*. Our results also suggest that the expression of the validated genes, except for *SLC16A3*, which showed slightly increased expression in TIL+ tumors, appears to be unaffected by TIL and PTIL status. Tao et al. clarified that a high lactate concentration in the tumor microenvironment leads to immunosuppression, which may mean that, despite the presence of immune cells in TIL+ tumors, their ability to combat tumor cells is limited due to increased *SLC16A3* expression [29]. This theory could suggest a potential role for *SLC16A3* as a therapeutic target in TIL+ tumors expressing *SLC16A3*. However, further studies are needed to confirm or refute this hypothesis.

Although our study primarily focused on genes involved in cancer-specific pathways such as hypoxia and EMT, our targeted transcriptome panel included genes relevant to the immune microenvironment, such as *CCL2*, *IFNG*, *IL6*, *TGFB1*, *TNF*, and *MKI67*. Differential expression of these genes might reflect changes in immune cell recruitment, inflammatory responses, and immune evasion mechanisms within colorectal cancer liver metastases. Future work utilizing whole transcriptome sequencing or extensive immune profiling, possibly coupled with RNA deconvolution approaches, would further clarify the relationship between identified gene expression changes and immune cell dynamics in the tumor microenvironment. Based on our results, each of the validated genes shows a varying degree of expression change between tumor and non-tumor samples. Moreover, the expression of almost all the selected genes was significantly altered in stage I of the disease compared to non-tumor samples, suggesting that they play a role in the early stages of carcinogenesis. Our findings also indicate that the expression of none of the validated genes is affected by the mutational status of the *KRAS* gene, which may imply that these genes are not involved in *KRAS+* CRC carcinogenesis. The findings related to the *P4HA1* and *SPP1* genes suggest they play important roles in the carcinogenesis, progression, and metastasis of CRC. This also supports their potential as biomarkers. For each of the validated genes, their potential as biomarkers has previously been proposed in the literature. Therefore, these genes warrant considerable attention in future research. Moreover, because a limitation of our study is the lack of matched primary and metastatic tumor samples from the same patients—which would enable a more direct assessment of tumor evolution—future studies including such paired samples would be highly valuable. Although our findings support *SPP1* and *P4HA1* as robustly differentially expressed genes in colorectal cancer, their precise biological roles in tumorigenesis and metastasis, as well as their clinical utility as biomarkers, remain to be determined. Future studies should include mechanistic investigations to elucidate their role in EMT and hypoxia-driven progression, and clinical validation in large, well-annotated cohorts to establish their independent prognostic or predictive value.

## 5. Conclusions

Our findings indicate that SPP1 and P4HA1 are consistently upregulated in colorectal cancer, supporting their consideration as candidate biomarkers. These results were validated in a larger cohort, strengthening their robustness. However, while our study establishes differential expression, their precise biological roles in tumorigenesis and metastasis, as well as their clinical utility as prognostic or predictive biomarkers, remain to be determined. Future studies should address these questions through functional and mechanistic experiments and evaluation in large, clinically annotated cohorts. If these hypotheses are confirmed, SPP1 and P4HA1 could contribute to improved diagnosis, prognosis, or treatment strategies in colorectal cancer.

## Figures and Tables

**Table 1:** Clinicopathological characteristics of individual patient groups: sex; age; clinical data; mutation statuses of *KRAS*, *PIK3CA*, *SMAD4*, *APC*, and *TP53* genes; and microsatellite instability. TILs—tumor-infiltrating lymphocytes; pTILs—peripheral tumor-infiltrating lymphocytes; MSI—microsatellite instability; MSS—microsatellite stability.

	Primary Tumors	Liver Metastases	Non-Tumor Controls
Patients	84	27	31
Female	39	7	15
Male	45	20	16
Age	69.5 ± 9.9	65.0 ± 7.9	72.8 ± 6.3
Clinical stage I/II/III/IV	15/25/26/7	0/0/0/27	
TIL-positive/-negative/n.a	13/27/44	12/1/14	
PTIL-positive/-negative/n.a	27/17/40	5/1/21	
*KRAS*-positive/-negative/n.a	24/28/32	11/14/2	
*PIK3CA*-positive/-negative/n.a	8/44/32	3/22/2	
*SMAD4*-positive/-negative/n.a	2/50/32	2/23/2	
*APC*-positive/-negative/n.a	21/31/32	10/15/2	
*TP53*-positive/-negative/n.a	24/28/32	13/12/2	
Mutations in driver genes 0/1/2/3 or more/n.a	7/18/17/10/32	3/7/13/2/2	
MSI/MSS/n.a	9/36/39	2/16/9	

**Table 2:** List of criteria for patient sample group allocation. MSI—microsatellite instability; *KRAS*—Kirsten rat sarcoma; *PIK3CA*—phosphatidylinositol-4,5-bisphosphate 3-kinase catalytic subunit alpha; *SMAD4—SMAD* family member 4; *TP53*—tumor protein p53; P/TILs—peripheral/tumor-infiltrating lymphocytes.

Tumors	Clinical stage III	Mutational status of *APC*
Primary tumors	Clinical stage IV	Mutational status of *TP53*
Liver metastases	MSI status	TIL-positive tumors
Control samples	Mutational status of *KRAS*	PTIL-positive tumors
Clinical stage I	Mutational status of *PIK3CA*	Tumors with 0, 1, 2, 3, and more mutations in driver genes
Clinical stage II	Mutational status of *SMAD4*	

## Data Availability

The data is contained within this article or its Supplementary Materials. All the raw data used in this study will be made available upon request. The R script used to generate the boxplots is provided at the following link: https://github.com/Emile-Jn/SPP1-CRC-boxplots accessed on 24 July 2025.

## Supplementary Materials

The following diagnostic information can be downloaded at https://www.mdpi.com/article/10.3390/cancers17193200/s1, Material S1: Gene list; Material S2: Overal Survival *SPP1*; Material S3: Overall survival *P4HA1*; Material S4: Supplementary Figures; Table S1: Clinicopathological characteristics and mutational status; Table S2: Differential gene expression; Table S3: Differentially expressed transcripts; Table S4: Statistical evaluations; Table S5; Statistical evaluation of matching samples.

## Abbreviations

The following abbreviations are used in this manuscript:

CRC	Colorectal cancer
DGE	Differential gene expression
DEGs	Differentially expressed genes
EMT	Epithelial-to-mesenchymal transition
PTIL	Peripheral tumor-infiltrating lymphocyte
TIL	Tumor-infiltrating lymphocyte
