# Supplementary material for: SPP1 as a Potential Stage-Specific Marker of Colorectal Cancer
Source: Cancers (Basel). 2025 Sep 30;17(19):3200. doi: 10.3390/cancers17193200 (PMC12523512; doi:10.3390/cancers17193200)
Supplement: Supplementary file 1 [file cancers-17-03200-s001.zip › Supplementary material S1.pdf]

ACD	BHLHE40	CCNE1	CDKN3	E2F4
ACVRL1	BIRC2	CCNF	CFLAR	EDN1
ADGRB1	BIRC3	CCNG1	CHEK1	EFNA1
ADM	BIRC5	CCNG2	CHEK2	EFNB2
ADORA2B	BLM	CCNH	CIB1	EGF
AHNAK	BMI1	CCNT1	CKS1B	EGR1
AKT1	BMP1	CD40	CKS2	EIF4EBP1
ALDOA	BNIP3	CD40LG	COL18A1	ENG
ALDOB	BNIP3L	CDC16	COL1A2	ENO1
ALDOC	BRCA1	CDC20	COL3A1	ENO2
ANAPC2	BRCA2	CDC25A	COL4A3	EPHB4
ANG	BRIP1	CDC25B	COL5A2	EPO
ANGPT1	C16orf13	CDC25C	CTGF	ERCC1
ANGPT2	CA9	CDC34	CTSA	ERCC2
ANGPTL4	CALD1	CDC6	CUL1	ERCC3
ANKRD37	CAMK2N1	CDH1	CUL2	ERCC4
ANXA2	CASP1	CDH2	CUL3	ERCC5
APAF1	CASP2	CDH5	CXCL12	ERCC6
APEX1	CASP3	CDK1	CXCL8	ERCC8
ATM	CASP6	CDK2	CYLD	ETS1
ATP6V1G2	CASP7	CDK4	DDB1	ETS2
ATR	CASP9	CDK5R1	DDB2	EXO1
AURKA	CAV2	CDK5RAP1	DDIT4	F3
AURKB	CCL2	CDK6	DESI1	FAM162A
BAX	CCNA2	CDK7	DFFA	FAS
BBC3	CCNB1	CDK8	DKC1	FASLG
BCCIP	CCNB2	CDKN1A	DLL4	FBP1
BCL2	CCNC	CDKN1B	DNAJC5	FBP2
BCL2A1	CCND1	CDKN2A	DSP	FBRSL1
BCL2L1	CCND2	CDKN2B	E2F1	FEN1
BCL2L11	CCND3	CDKN2D	E2F3	FGF1

FGF2	ITGA5	MKI67	NUDT13	PKLR
FGFBP1	ITGAV	MLH1	OBFC1	PKM
FIGF	ITGB3	MLH3	OCLN	PLAU
FLT1	JAG1	MMP14	ODC1	PLG
FN1	JMJD6	MMP2	OGG1	PLK1
FOXC2	KDR	MMP3	P4HA1	PMS1
G6PC	KIAA0586	MMP9	PARP1	PMS2
GADD45A	KNTC1	MMS19	PARP2	PNKP
GAR1	KPNA2	MNAT1	PARP3	POLB
GNG11	KRT19	MPG	PC	POLL
GPI	LDHA	MRE11A	PCK1	POT1
GSC	LDHB	MSH2	PCK2	PPIE
GTSE1	LEP	MSH3	PDHA1	PPIL2
HGF	LGALS3	MSH6	PDK1	PRKAA1
HIF1A	LIG1	MSN	PDK2	PRKAB1
HK1	LIG3	MST1R	PDK3	PRKDC
HK2	LIG4	MTOR	PDK4	PTGES3
HK3	LOX	MXI1	PF4	PTGS2
HMOX1	MAD2L1	NAMPT	PFKFB1	RAD1
HSP90AA1	MAD2L2	NBN	PFKFB2	RAD17
HUS1	MAP3K1	NDRG1	PFKFB3	RAD18
ID1	MAP3K2	NEIL1	PFKFB4	RAD21
IER3	MCL1	NEIL3	PFKL	RAD23A
IFNG	MCM2	NHP2	PFKM	RAD23B
IGF1	MCM3	NOL3	PGAM2	RAD50
IGF1R	MCM4	NOP10	PGF	RAD51
IGFBP3	MCM5	NOS3	PGK1	RAD51B
IGFBP4	MDM2	NOTCH4	PGK2	RAD52
IL1B	MET	NRP1	PGM1	RAD54L
IL1RN	MGMT	NRP2	PIM1	RAD9A
IL6	MIF	NTHL1	PINX1	RAP1A

RB1
RBBP8
RBL1
RBL2
REV1
RFX1
RGS2
RIF1
RNF8
RPA1
RPA3
RTEL1
RUNX2
RUVBL2
SERPINE1
SERPINF1
SERTAD1
SIRT6
SKP2
SLC16A3
SLC2A1
SLC2A3
SLC2A4
SMUG1
SNAI1
SNAI2
SNAI3
SOX10
SP1
SPARC
SPATA2
SPP1
STEAP1
STK11
STMN1
SYCP2
TCF4
TDG
TEK
TEP1
TERF1
TERF2
TERF2IP
TERT
TFDP1
TFDP2
TFPI2
TFRC
TGFB1
TGFBR1
THAP3
THBS1
THBS2
TIMP1
TIMP2
TINF2
TMEFF1
TMEM132A
TNF
TNFRSF10A
TNFRSF11B
TNFRSF1A
TNKS
TNKS2
TP53
TP53BP1
TP73
TPI1
TRAF2
TSPAN13
TWIST1
TXNIP
UNG
VCAN
VDAC1
VEGFA
VEGFC
VIM
VPS13A
WEE1
WNT5A
WNT5B
XAB2
XIAP
XPA
XPC
XRCC1
XRCC2
XRCC3
XRCC4
XRCC5
XRCC6
ZBTB22
ZNF446
_GDC_CONTROL_06_
_GDC_CONTROL_07_
_GDC_CONTROL_14_
_GDC_CONTROL_21_
_GDC_CONTROL_24_
_GDC_CONTROL_32
