# Supplementary material for: SPP1 as a Potential Stage-Specific Marker of Colorectal Cancer
Source: Cancers (Basel). 2025 Sep 30;17(19):3200. doi: 10.3390/cancers17193200 (PMC12523512; doi:10.3390/cancers17193200)
Supplement: Supplementary file 1 [file cancers-17-03200-s001.zip › Supplementary material S4.pdf]

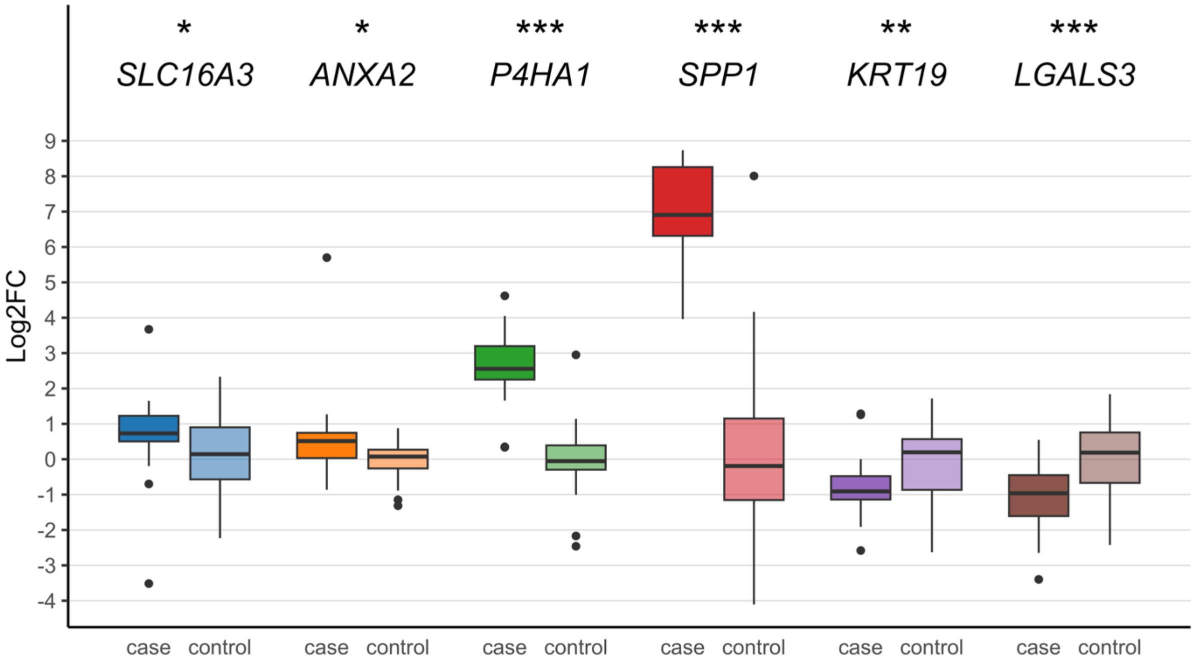

Supplementary Figure S1: Boxplots showing fold change difference of *SLC16A3*, *ANXA2*, *P4HA1*, *SPP1*, *KRT19* and *LGALS3* genes in liver metastases (case) compared to non-tumor (control) tissues (*p < 0.05, **p < 0.01; ***p < 0.001).

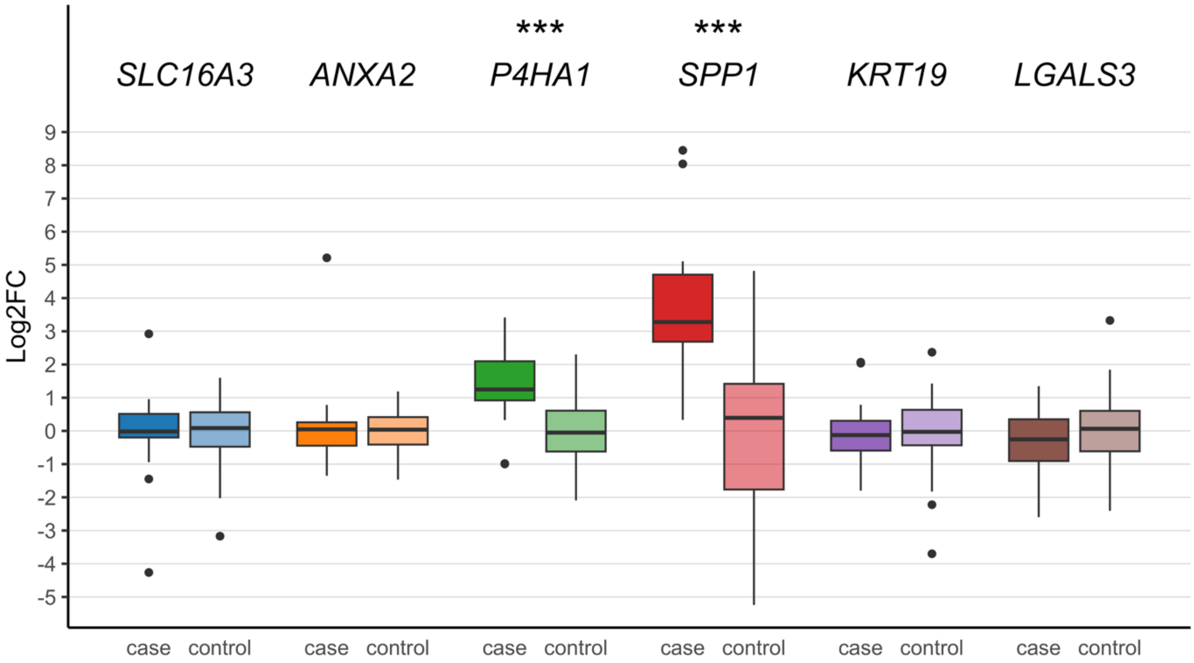

Supplementary Figure S2: Boxplots showing fold change difference of *SLC16A3*, *ANXA2*, *P4HA1*, *SPP1*, *KRT19* and *LGALS3* genes in liver metastases (case) compared to primary tumor (control) samples (***p < 0.001).

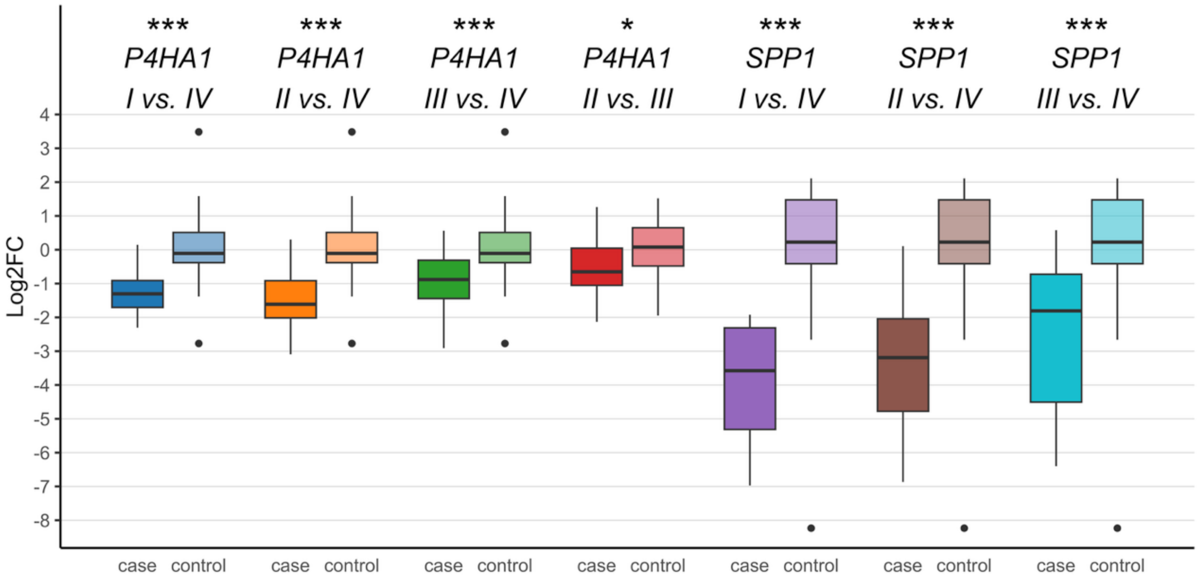

Supplementary Figure S3: Boxplots comparing the change in expression of *P4HA1* and *SPP1* genes between distinct CRC stages. The specific pairwise comparisons between CRC stages are indicated within the figure (*p < 0.05, ***p < 0.001).

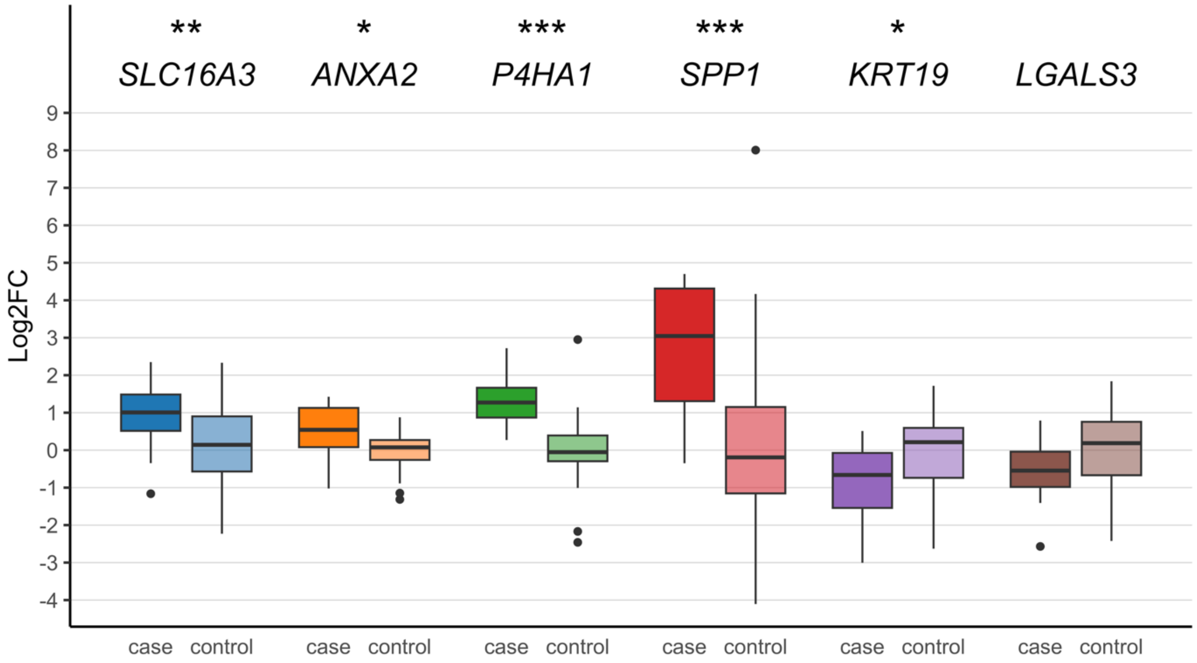

Supplementary Figure S4: Boxplots showing fold change difference of *SLC16A3*, *ANXA2*, *P4HA1*, *SPP1*, *KRT19* and *LGALS3* genes in stage I samples (case) compared to control non-tumor (control) samples. The log_2FC of the control samples represents a value close to 0 (*p < 0.05, **p < 0.01; ***p < 0.001).

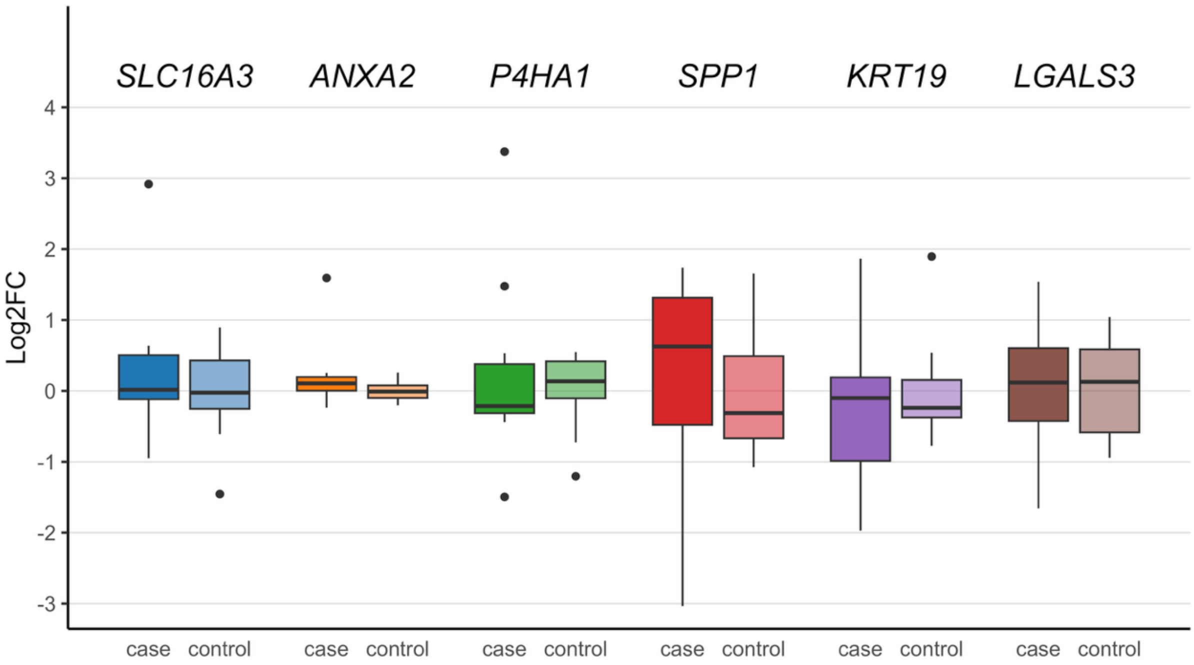

Supplementary Figure S5: Boxplots showing fold change difference of *SLC16A3*, *ANXA2*, *P4HA1*, *SPP1*, *KRT19* and *LGALS3* genes in *KRAS*+ metastases compared to *KRAS*- metastases. The log_2FC of the *KRAS*- metastatic samples represents a value close to 0.
