# Supplementary figures and images for: SPP1 as a Potential Stage-Specific Marker of Colorectal Cancer
Source: Cancers (Basel). 2025 Sep 30;17(19):3200. doi: 10.3390/cancers17193200 (PMC12523512; doi:10.3390/cancers17193200)

# Overall Survival

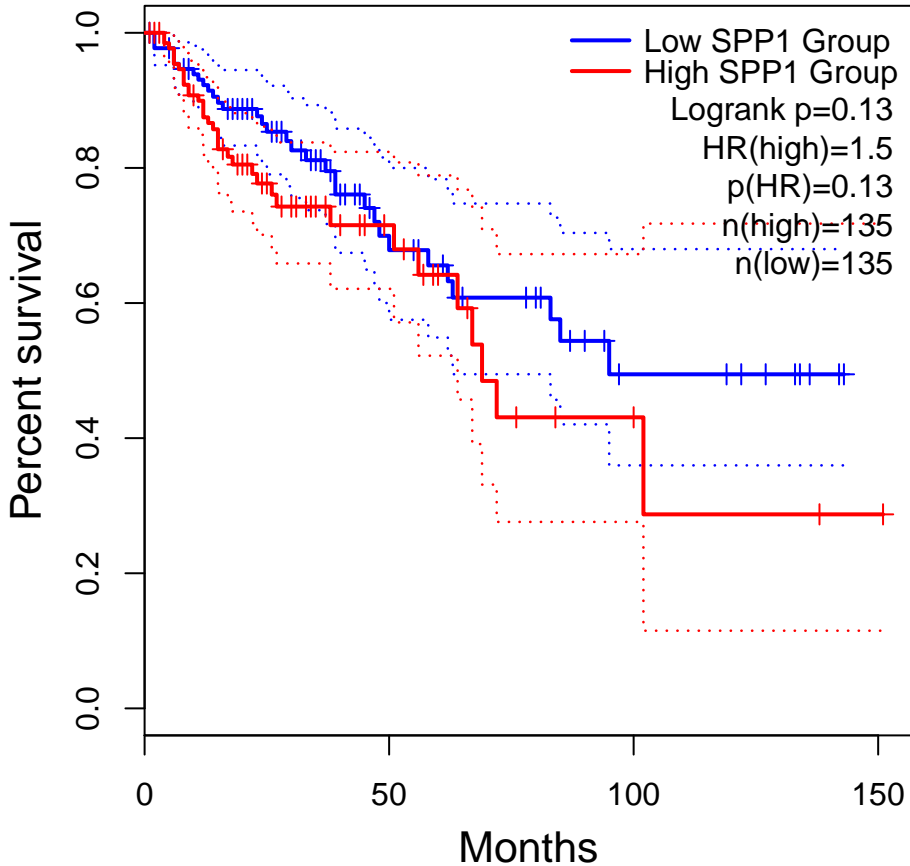

Supplement: Supplementary file 1 [file cancers-17-03200-s001.zip › Supplementary material S2_SPP1_survival.pdf]

# Overall Survival

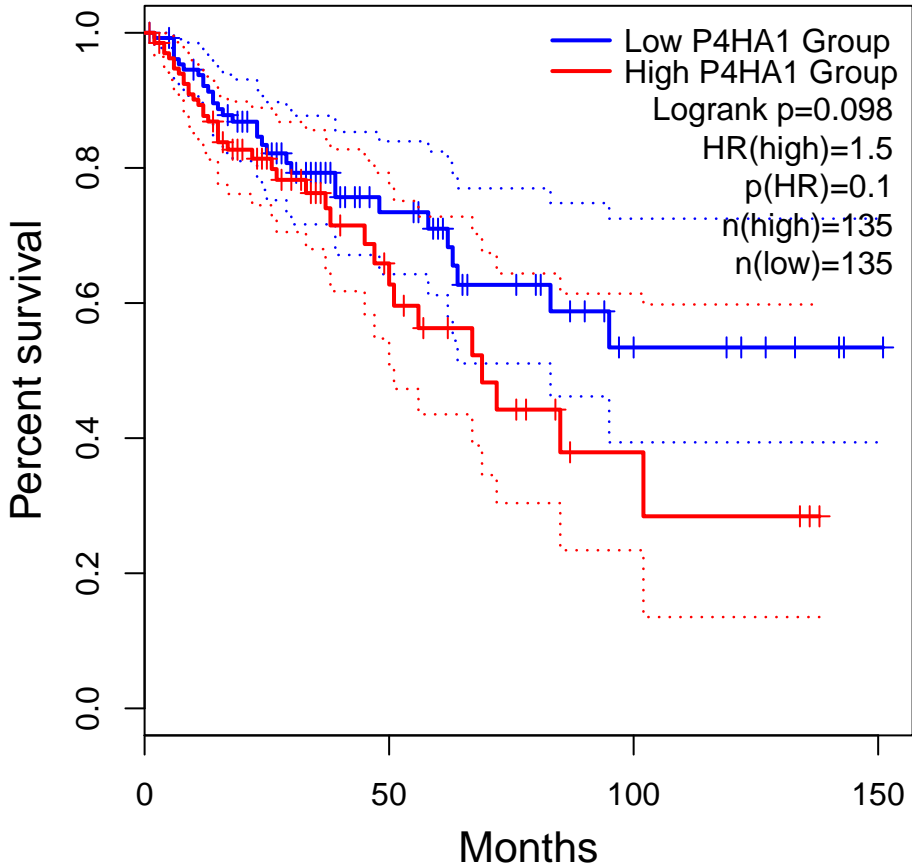

Supplement: Supplementary file 1 [file cancers-17-03200-s001.zip › Supplementary material S3_P4HA1_survival.pdf]
